# Extended Berry Curvature Tail in Ferromagnetic Weyl Semimetals NiMnSb and PtMnSb

**DOI:** 10.1002/advs.202404495

**Published:** 2024-06-18

**Authors:** Sukriti Singh, Ana García‐Page, Jonathan Noky, Subhajit Roychowdhury, Maia G. Vergniory, Horst Borrmann, Hans‐Henning Klauss, Claudia Felser, Chandra Shekhar

**Affiliations:** ^1^ Max Planck Institute for Chemical Physics of Solids 01187 Dresden Germany; ^2^ Donostia International Physics Center Donostia‐San Sebastian 20018 Spain; ^3^ Institute for Solid State and Materials Physics Technische Universität Dresden 01069 Dresden Germany; ^4^ Department of Chemistry Indian Institute of Science Education and Research Bhopal Bhopal 462066 India; ^5^ Present address: Institute of Solid‐State Physics Vienna University of Technology Vienna 1040 Austria; ^6^ Present address: Max Planck Institute for Solid State Research 70569 Stuttgart Germany

**Keywords:** anomalous Hall effect, Berry curvature, half Heusler compound, Weyl semimetals

## Abstract

Heusler compounds belong to a large family of materials and exhibit numerous physical phenomena with promising applications, particularly ferromagnetic Weyl semimetals for their use in spintronics and memory devices. Here, anomalous Hall transport is reported in the room‐temperature ferromagnets NiMnSb (half‐metal with a Curie temperature (*T*_C_) of 660 K) and PtMnSb (pseudo half‐metal with a *T*_C_ of 560 K). They exhibit 4 µ_B_/f.u. magnetic moments and non‐trivial topological states. Moreover, NiMnSb and PtMnSb are the first half‐Heusler ferromagnets to be reported as Weyl semimetals, and they exhibit anomalous Hall conductivity (AHC) due to the extended tail of the Berry curvature in these systems. The experimentally measured AHC values at 2 K are 1.8 × 10^2^ Ω^−1 ^cm^−1^ for NiMnSb and 2.2 × 10^3^ Ω^−1^ cm^−1^ for PtMnSb. The comparatively large value between them can be explained in terms of the spin‐orbit coupling strength. The combined approach of using ab initio calculations and a simple model shows that the Weyl nodes located far from the Fermi energy act as the driving mechanism for the intrinsic AHC. This contribution of topological features at higher energies can be generalized.

## Graphical abstract

Anomalous Hall conductivity (AHC) is strongly influenced by topological property type and its proximity to the Fermi energy (*E*_F_). It is generally assumed that the topology near to the *E*_F_ has a greater impact on the AHC than the topology further away from the *E*_F_. However, the new discovery, as reported in the manuscript, demonstrates that the AHC is determined solely by Berry curvature (BC), even when it is located far away from the *E*_F_ (right panel). This effect is named as *extended Berry curvature tail effect*, where the source of BC, i.e., Weyl node does not necessarily have to be close to *E*_F_. The effect can spread beyond *E*_F_ as long as the topological band is clean (left panel: orange and gree bands)and there is no interference from other trivial bands.

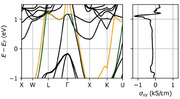

## 1 Introduction

The anomalous Hall effect (AHE) is an intriguing transport phenomenon observed in ferromagnets, resulting from the combination of broken time reversal symmetry (TRS) and spin–orbit interactions.^[^ ^1^ ^]^ TRS is broken due to the spontaneous magnetization, which affects the motion of electrons. The AHE was initially discovered in ferromagnetic materials and was first explained as an intrinsic effect by Karplus and Luttinger.^[^ ^2^ ^]^ In addition to the ordinary Hall effect, which is the result of the Lorentz force experienced by charge carriers, an additional term known as the AHE term is included in ferromagnetic materials due to the broken TRS. Such systems with longitudinal currents, even without a magnetic field, are characterized by a scaling with the field‐dependent magnetization. In contrast, the ordinary Hall effect scales linearly with the applied magnetic field. The AHE involves two major mechanisms: an extrinsic mechanism resulting from spin–orbit scattering (skew), and an intrinsic mechanism resulting from the Berry curvature (BC) of non‐trivial electronic bands.^[^ ^3^ ^]^ In the recent years, there has been a renewed interest in the AHE driven by BC due to the discovered relationship between electron motion (anomalous velocity) and the Berry phase. The anomalous Hall conductivity (AHC) can be obtained by integrating the BC for all occupied states in the momentum space.^[^ ^4^ ^]^ To observe the effect of BC‐induced transport in real materials, it is necessary to assume that the topological features such as Weyl nodes are near the Fermi energy (*E*_F_). This study demonstrates that BC induced AHC can be significant even when the Weyl nodes are located far from the *E*_F_. This is due to the ability of the BC from these nodes to be dragged to the *E*_F_.

The Heuslers are a very broad family and various ferromagnetic full‐Heusler candidates have been investigated in search of the BC‐driven AHE. Co‐ and Fe‐based full‐Heusler compounds such as Co_2_MnAl,^[^ ^5^ ^]^ Co_2_MnGa,^[^ ^6^ ^]^ Fe_2_NiGa, and Fe_2_CoAl,^[^ ^7^, ^8^ ^]^ among many others, have been thoroughly investigated in recent studies.^[^ ^9^, ^10^ ^]^ However, the research on ferromagnetic half‐Heusler compounds is still in its early stages. The only few antiferromagnetic half‐Heusler RPtBi (R = Gd, Nd, Tb, Ho) compounds have been identified for BC‐driven AHE.^[^ ^11^, ^12^, ^13^ ^]^ However, half‐Heusler half‐metallic ferromagnets, which are rare, have not been well investigated. Therefore, it is crucial to comprehend the origin of the AHE in the half‐Heusler ferromagnetic family from a fundamental standpoint. Several ferromagnetic half‐Heusler compounds have been predicted theoretically, but only a few have been experimentally realized.^[^ ^14^, ^15^, ^16^, ^17^, ^18^, ^19^, ^20^, ^21^, ^22^ ^]^ NiMnSb and PtMnSb are well‐known half‐Heusler ferromagnets with known electronic band structures and magnetic properties.^[^ ^21^, ^22^, ^23^, ^24^, ^25^, ^26^, ^27^, ^28^, ^29^, ^30^ ^]^ These compounds are still the center of attention for the nonvolatile mass storage memory purposes and for various spintronic applications due to their unique characteristics, such as in room‐temperature ferromagnetism,^[^ ^31^ ^]^ spin torque,^[^ ^28^, ^32^ ^]^ and large spin polarization.^[^ ^33^, ^34^, ^35^ ^]^

**Figure 1:**
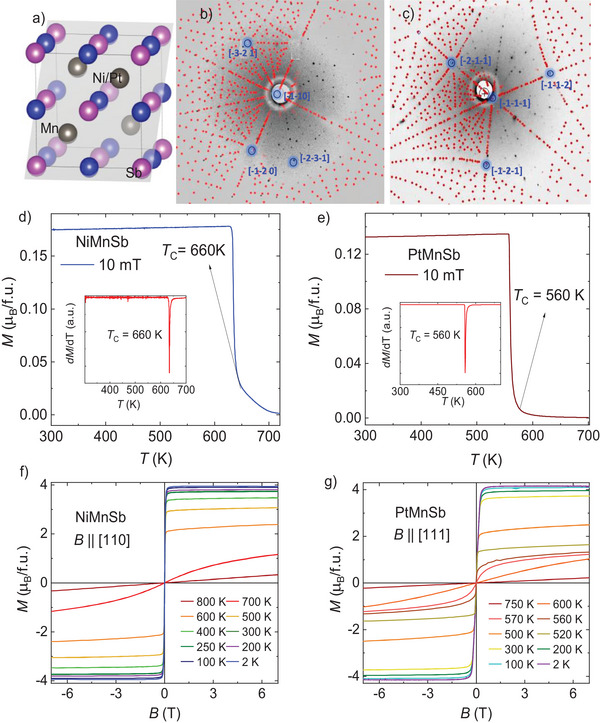
Structure, Laue diffraction, and magnetization. a) Schematic crystal structure of a half‐Heusler system with an *F*‐43*m* (216) space group. Laue XRD for b) NiMnSb and c) PtMnSb. d,e) Temperature dependent magnetization curves measured at an applied magnetic field of 10 mT showing very high Curie temperatures of *T*_C_ = 660 and 560 K for NiMnSb and PtMnSb, respectively. First order derivative is taken to find more accurate *T*_C_ values, as shown in the inset. f,g) Isothermal magnetization curves measured with a magnetic field applied parallel to [110] and [111] for NiMnSb and PtMnSb, respectively.

Here, we report the anomalous Hall behavior of NiMnSb and PtMnSb. These compounds exhibit very high Curie temperature (*T*_C_) values above room temperature (specifically 660 K and 560 K, respectively). Both compounds crystallize in the non‐centrosymmetric cubic structure of the MgAgAs‐type (*C*1*_b_*) space group *F*‐43*m* (216) with three interpenetrating *fcc* lattices, each occupied by Ni (Pt), Mn, and Sb atoms, as shown in **Figure** **1**a. The lattice parameters are 5.93 Å for NiMnSb and 6.20 Å for PtMnSb. Figures 1b,c show the Laue X‐Ray diffraction patterns of NiMnSb and PtMnSb crystals, respectively, oriented along the [110] and [111] directions. Both compounds exhibit non‐saturated positive magnetoresistance (MR) values that become negative with increasing temperature. AHC values of 180 and 2200 Ω^−1 ^cm^−1^ at 2 K are observed in NiMnSb and PtMnSb, respectively. In NiMnSb, the intrinsic contribution is 135 Ω^−1^ cm^−1^, while in PtMnSb it is 410 Ω^−1^ cm^−1^. Ab‐initio calculations shows that the Weyl nodes, which are located far from the *E*_F_, act as the driving mechanism for the intrinsic AHC.

## 2 Results and Discussion

The preferred crystal growth orientation of the compounds is [111] when they are grown via the flux method, and we therefore performed transport measurements with an applied magnetic field in the direction [111]. However, NiMnSb was additionally grown using a laser floating zone (LFZ) technique. The crystal was oriented along [110], and all measurements were performed with an applied magnetic field along [110]. Figures 1d,e show the temperature‐dependent magnetization obtained for a 10 mT field, and a sharp jump is observed around the transition temperature. Both the compounds NiMnSb and PtMnSb exhibit very high *T*_C_ of 660 and 560 K, respectively. To determine more accurate *T*_C_ values, the first‐order derivative of magnetization (d*M*/d*T*) is taken as shown in the insets of Figures 1d,e. Figures 1f,g show the field‐dependent magnetization obtained at various temperature in the range of 2 to 800 K, revealing that the saturation magnetization values are 3.95 and 4.12 µ_B_/f.u. at 2 K for NiMnSb and PtMnSb, respectively. As the temperature increases, the saturation magnetization decreases, and as *T*_C_ is approached, the saturation magnetization is achieved at a relatively higher magnetic field.

**Figure 2:**
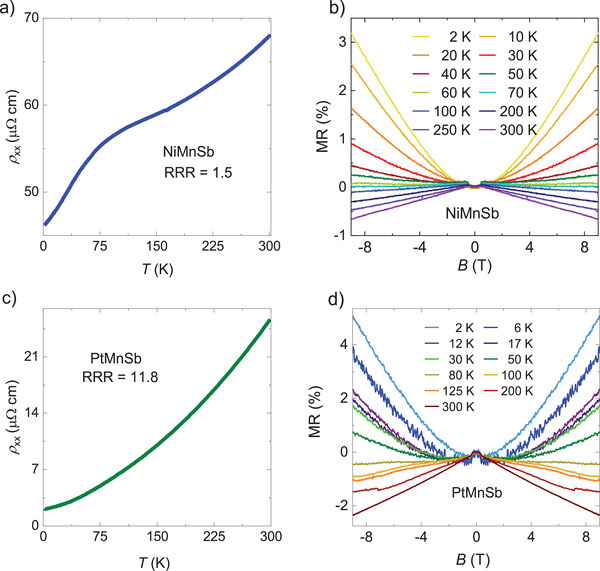
Electrical transport measurements. a,c) Longitudinal resistivity *ρ*_xx_ measured for NiMnSb and PtMnSb, respectively. b,d) Magnetoresistance measured in a field ranging from −9 to +9 T for NiMnSb and PtMnSb, respectively.

**Figures** **2**a,c show the temperature‐dependent longitudinal resistivities of NiMnSb and PtMnSb, respectively. NiMnSb has a residual resistivity ratio (RRR) of 1.5, while PtMnSb has an RRR nearly seven times larger than NiMnSb, and it is 11.8. The broad feature in the longitudinal resistivity of NiMnSb shown in Figure 2a was also previously observed, and it was claimed to result from a high Mn content.^[^ ^24^ ^]^ Figures 2b,d show the transverse magnetoresistance of NiMnSb and PtMnSb, respectively, which were measured by varying the magnetic field from +9 to −9 T at various temperatures between 2 to 300 K. NiMnSb and PtMnSb exhibit positive magnetoresistance (MR) of 3.3% and 4.3% at a low temperature of 2 K, respectively, whereas at high temperatures, these values become negative (−0.7% and −2.4% at 300 K, respectively). Magnetic compounds typically exhibit this trend, as the applied magnetic field suppresses the occurrence of spin‐disorder scattering.

**Figure 3:**
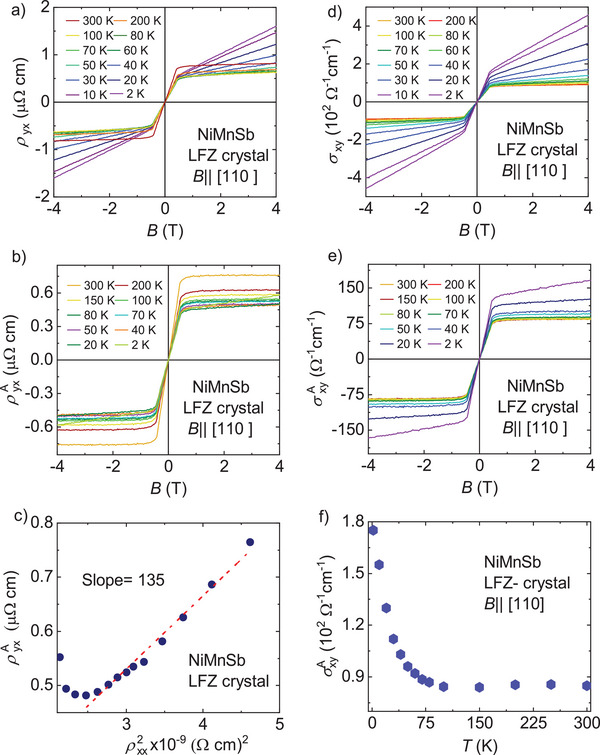
Anomalous Hall measurements of NiMnSb. a) Field‐dependent Hall resistivity measured with an applied magnetic field along the (110) direction. b) Reduced anomalous Hall resistivity as a function of the magnetic field obtained by subtracting the ordinary Hall resistivity. c) Plot of $\rho_{yx}^{A}$ versus $\rho_{xx}^{2}$ used to determine the intrinsic contributions. d) Field‐dependent Hall conductivity measured with an applied magnetic field along the (110) direction. e) AHC as function of the magnetic field. f) Temperature‐dependent AHC.

**Figure** **3**a shows the Hall resistivity (*ρ*_yx_) measured for the NiMnSb LFZ crystal with the field oriented along the crystallographic axis [110] in the temperature range of 2 to 300 K. In ferromagnetic materials, *ρ*_yx_ is generally defined as *ρ*_yx_ = (*R*_0_ *B* + *R*_S_ *M*), where *R*_0_ and *R*_S_ are the ordinary and anomalous Hall coefficients, respectively, and *B* and *M* are the magnetic field and magnetization, respectively. The equation includes two contributions from Hall effect. The first term represents the ordinary Hall effect, which results from the Lorentz force acting on the charge carriers when magnetic field is applied. The second term, known as the AHE, is unique to magnetic samples. In NiMnSb and PtMnSb, the contribution from the Hall effect is significant at low temperatures and decreases as the temperature increases. Additionally, the MR value becomes positive with increasing temperature. In the case of NiMnSb, *R*_0_ increases in magnitude as the temperature decreases (Figure S1a, Supporting Information). *R*_0_ is calculated using the slope of the Hall resistivity at high field. At the 300 K, the value of *R*_0_ is 1.4 × 10^4^ cm^3^C^−1^, while at 2 K, it increases to 26 × 10^4^ cm^3^ C^−1^. The sign of *R*_0_ depicts the type of charge carrier involved in transport. In the case of NiMnSb, the majority charge carriers are holes. Figure 3d shows the variation of Hall conductivity with the magnetic field, which is calculated using the equation ( σ_*xy*_ = $\frac{\rho_{yx}}{\rho_{yx}^{2}+\rho_{xx}^{2}})$. Figure 3b shows the anomalous Hall resistivity ($\rho_{yx}^{A}$) varying with the magnetic field ranging from +9 to −9 T (shown only in the range of +4 to −4 T for better presentation), which is deduced by subtracting the ordinary Hall contribution. Here, the ordinary Hall contribution has been subtracted by linear fit in the high magnetic field region. Figure 3e shows the AHC variation with the magnetic field, which is evaluated using $\rho_{yx}^{A}$. At the 300 K, the value of AHC is ≈90 Ω^−1^ cm^−1^, which increases to 1.8 × 10^2^ Ω^−1^ cm^−1^ at 2 K. The AHC remains almost constant at 90 Ω^−1^cm^−1^ after 75 K. Using the standard scaling law, the intrinsic contribution can be experimentally estimated by the slope of the linear fit of $\rho_{yx}^{A}$ versus $\rho_{xx}^{2}$, which is 1.35 × 10^2^ Ω^−1^ cm^−1^, as shown in Figure 3c.^[^ ^34^ ^]^ Figure 3f shows the temperature dependence of $\sigma_{xy}^{A}$ for NiMnSb. The value of $\sigma_{xy}^{A}$ decreases by nearly 50% when the temperature reaches 70 K and becomes nearly constant until 300 K. In the case of the flux crystals shown in Figure S5d (Supporting Information), the decrease in $\sigma_{xy}^{A}$ is only 18% from 1.8 to 300 K. Additionally, Hall measurements were performed on the crystal grown using the flux method, and the results are shown in Figures S3–S6 (Supporting Information), indicating similar results to the crystal grown using the LFZ technique. For example, the intrinsic contributions of AHC in the flux and LFZ crystals are 1.1 × 10^2^ Ω^−1^ cm^−1^ (Figure S5d, Supporting Information) and 1.35 × 10^2^ Ω^−1^ cm^−1^, respectively, which are almost same. This provides an indication that the evaluation of the intrinsic contributions is valid, as the value of $\sigma_{xy}^{A}$ does not differ greatly. However, differences in the defects and disorder may be present due to use of different crystal growth methods.

**Figure 4:**
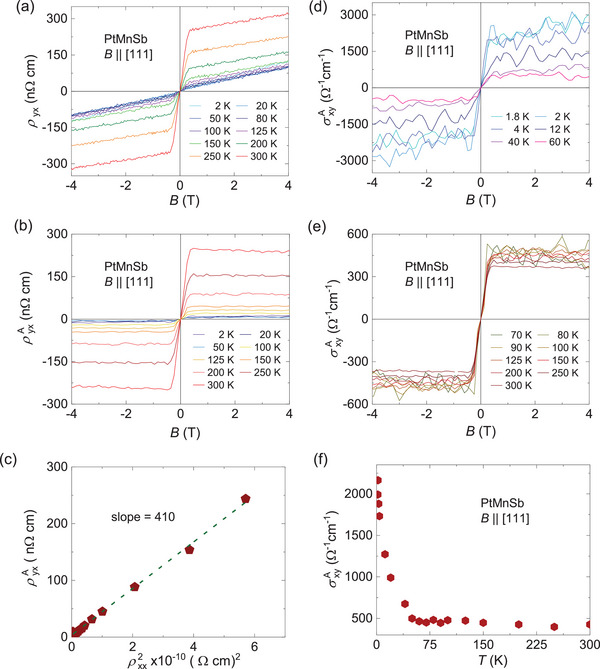
Anomalous Hall measurements of PtMnSb. a) Field‐dependent Hall resistivity measured with an applied magnetic field along the (111) direction. b) Reduced anomalous Hall resistivity as a function of the magnetic field obtained by subtracting the ordinary Hall resistivity. c) Plot of $\rho_{yx}^{A}$ versus $\rho_{xx}^{2}$ used to determine the intrinsic contributions, wherein the slope of the linear fitting provides the intrinsic contributions. AHC as a function of the magnetic field at d) low and e) high temperatures. f) AHC as function of temperature.

In case of PtMnSb, the transport behavior is similar to that of NiMnSb. The transport properties were measured with the field oriented along the crystallographic axis [111], as shown in **Figure** **4**. The value of the Hall resistivity is in the order of 1 nΩ cm, as shown in Figure 4a. At 300 K, this value reaches 320 nΩ cm at an applied magnetic field of 4 T and decreases to 94 nΩ cm at 2 K. At low temperatures, the ordinary Hall contribution dominates, and the anomalous Hall contribution appears to be absent. Upon examining the 2 K Hall resistivity data shown in Figure S7 (Supporting Information), a clear change in slope is evident at 0.3 T, indicating the presence of anomalous Hall contributions. To obtain the anomalous Hall resistivity, the linear fit at high magnetic field was subtracted, as shown in Figure 4b. Figures 4d,e show the field‐dependent $\sigma_{xy}^{A}$ values obtained at low and high temperatures, respectively. At 2 K, the value of $\sigma_{xy}^{A}$ is 2164 Ω^−1^ cm^−1^, which decreases to 450 Ω^−1^ cm^−1^ at 70 K. Interestingly, the value of $\sigma_{xy}^{A}$ at 300 K is 427 Ω^−1^ cm^−1^, which illustrates that $\sigma_{xy}^{A}$ remains nearly constant after 70 K as the temperature increases, similar to NiMnSb. The majority charge carriers for PtMnSb are holes, as indicated by the Hall coefficient *R*_0_, as shown in Figure S1b (Supporting Information). To estimate the BC contribution, a linear fit is performed for the plot of $\rho_{yx}^{A}$ versus $\rho_{xx}^{2}$, as shown in Figure 4c. The intrinsic contribution is determined to be 410 Ω^−1^ cm^−1^, which accounts for only 18% of the total $\sigma_{xy}^{A}$ value observed at 2 K. However, the primary goal of this study is to gain an understanding of the effect of the extended BC tail on the electrical transport properties. Figure 4f shows the temperature‐dependent AHC, where the value of $\sigma_{xy}^{A}$ decreases by ≈ 80% when the temperature reaches 70 K, and becomes nearly constant up to room temperature. In typical magnetic materials, a decrease in $\sigma_{xy}^{A}$ is expected as the temperature approaches *T*_C_; however, in the investigated system, $\sigma_{xy}^{A}$ decreases at T = 0.11 *T*_C_. This decrease in $\sigma_{xy}^{A}$ is consistent with the decrease in the Hall coefficient shown in Figure S1a (Supporting Information). The NiMnSb and PtMnSb results show that the AHC decreases exponentially before approaching *T*_C_. The sudden decrease in AHC in both the compounds may be attributed to the thermal collapse of the spin polarization in these half‐metallic ferromagnets.^[^ ^37^ ^]^ This affects only the extrinsic part of $\sigma_{xy}^{A}$, while the intrinsic part remains unchanged. The experimental extrinsic contribution $\sigma_{xy}^{A}$ to the case of PtMnSb indicates that the skew scattering contribution dominates in the AHC below 30 K with *σ*_*xx*_ > 10^5^ Ω^−1^cm^−1^, which is the clean limit. The magnitude of the *σ*_*xx*_ has been verified in different piece of crystals and it is reproducible, as shown in Figure S9 (Supporting Information). In the case of NiMnSb, *σ*_*xx*_ is on the order of 10^4^ Ω^−1^cm^−1^, which lies in the intrinsic regime, where BC contribution is more expected to dominate.^[^ ^1^ ^]^ More examples are given in the Figure S10 (Supporting Information) including our present compounds, where a crossover between intrinsic and skew scattering regimes is mentioned.

**Figure 5:**
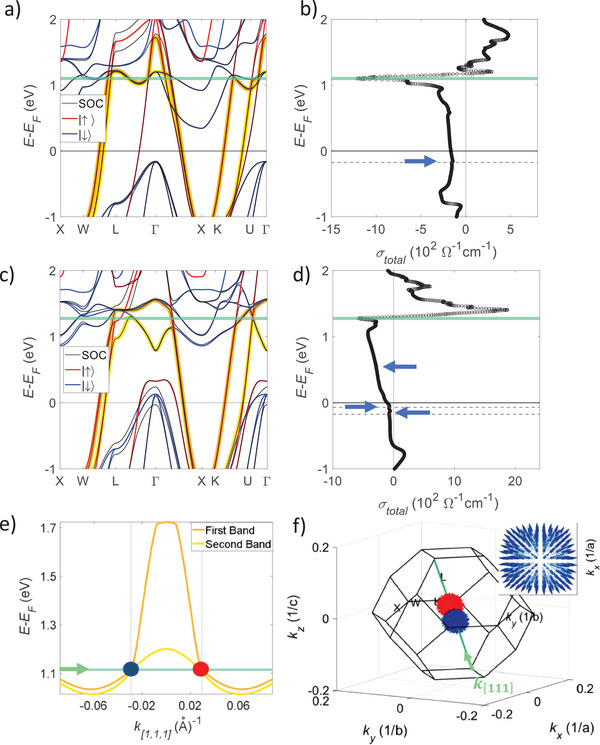
Band structure and calculated AHC values a, c) Electronic band structures of NiMnSb and PtMnSb, respectively, with SOC (black solid lines) and without SOC (spin‐up in red solid lines; spin‐down in blue solid lines). The two high velocity bands are highlighted in yellow and orange, respectively. b, d) Energy dependence of the calculated intrinsic AHC. The Weyl nodes close to the *E*_F_ are indicated by blue arrows, which cause only small bumps in the AHC. Nevertheless, the AHC is dominated by Weyl nodes from crossings of the high velocity lines far above from the Fermi level (denoted by the green solid line). Two such crossings for NiMnSb in the [111] are shown in e), giving rise to a pair of Weyl nodes f) that act as sources of the BC (inset).

The BC can be seen as a correction to the single‐band description of materials because it represents the residual interactions between adjacent bands. When two bands cross non‐trivially, topological structures such as Weyl nodes (0‐dimensional) or nodal lines (1‐D) appear, leading to a finite total BC in the material. When these crossings occur near the Fermi level (*E*_F_), the transport properties of the material are deeply affected, such as the AHC measured in this study. Conversely, the net BC of the material is finite only under certain circumstances, such as spin–orbit coupling (SOC) combined with the TRS breaking. Therefore, the band structure of the compounds, which depends on the presence of crystal symmetries, is the origin of the BC. Therefore, the band structures of both NiMnSb and PtMnSb were obtained (**Figures** **5**a,c) in the high symmetry line X‐W‐L‐Γ‐X‐K‐U. In both the compounds the spin‐down channel forms a gapped state, although for PtMnSb, the *E*_F_ value is slightly below this gap. The half‐metallic and pseudo half‐metallic behaviors of NiMnSb and PtMnSb, respectively, can be seen in Figures 5a,c. In the latter case, a spin‐down pocket appears at *Γ* in the *E*_F_, reducing the spin‐up polarization of the compound.^[^ ^38^ ^]^ The spin‐up bands close this gap with a strong dispersion. In both the compounds, the AHC is a product of the SOC combined with magnetism, allowing a non‐vanishing net BC to appear. This can be directly observed in Figure 5. The hybridization of bands from different spin channels obtained when the SOC is included (Figures 5a,c) leads to a finite AHE (Figures 5b,d).

It is important to note that the calculated AHC values of NiMnSb are relatively constant in an energy window of −0.5 to 0.5 eV around *E*_F_, which allows a straightforward comparison with the experimental results. As mentioned above, both the values are in very good agreement, regardless of the synthesis procedure. For PtMnSb, however, the AHC is very much energy dependent around *E*_F_, making a comparison rather difficult.

To understand the origin of the AHE in these compounds, we need to discuss the band structure in more detail. It is known that the Weyl points close to the *E*_F_ generate a peak in the AHC,^[^ ^39^ ^]^ and these peaks become more pronounced as the distance between the two Weyl nodes of a pair increases.^[^ ^40^ ^]^ In both of the compounds considered in this study, Weyl crossings are present close to the *E*_F_ (see blue arrows in Figures 5b,d) at the following energies: 170 meV below the *E*_F_ for NiMnSb, and −70, −46, and 624 meV from the *E*_F_ for PtMnSb, resulting in small bumps in the AHC curves of both compounds (Figures 5b,d and Tables S2 and S3, Supporting Information). Nevertheless, these Weyl nodes are very close to each other in both the materials, with a distance of less than 0.01 Å^−1^ and thus have a negligible influence on the AHC (for a detailed analysis of the contributions see Figures S11 and S12, Supporting Information). Therefore, an additional mechanism, different from the one resulting from the presence of Weyl nodes close to the *E*_F_, must be responsible for the high AHC values found in both the experiments and the theoretical calculations. By visually inspecting the band structures of these systems (Figures 5a,c), it can be seen that both exhibit two bands that are very close to each another with a high velocity at the *E*_F_ (as shown in yellow in Figures 5a,c). Interestingly, these two bands cross at a distance far from *E*_F_, resulting in the formation of three pairs of Weyl nodes at 1.26 eV for PtMnSb and seven pairs at 1.1 eV for NiMnSb above the *E_F_*. Moreover, these Weyl nodes not only give rise to the expected large peak of the AHC at the energy level of such crossings (see green lines in Figures 5b,d) but also appear to be responsible for the finite AHC values present in both the compounds. In the present study, we propose that these two combined features (i.e., the existence of bands crossing the *E*_F_ with a large slope and these two bands form Weyl nodes far away from the *E*_F_) can lead to finite AHC values. The expanded view of two such crossings along [111] for NiMnSb and corresponding a pair of Weyl nodes in the Brillouin zone are shown in Figures 5e and  5f, respectively.

To better understand this effect, we use a simple two‐band tight‐binding toy model derived from^[^ ^39^, ^40^, ^41^ ^]^:

(1) $$H\;\left(\vec{k}\right)=\;\left[\begin{array}{cc} B_{1}\left(\cos k_{x}+\cos k_{y}+\cos k_{z}\right)+M_{1} & A\left(\sin k_{x}-i\sin k_{y}\right)\\ A(\sin k_{x}+i\sin k_{y}) & B_{2}\left(\cos k_{x}+\cos k_{y}+\cos k_{z}\right)+M_{2} \end{array}\right]$$

**Figure 6:**
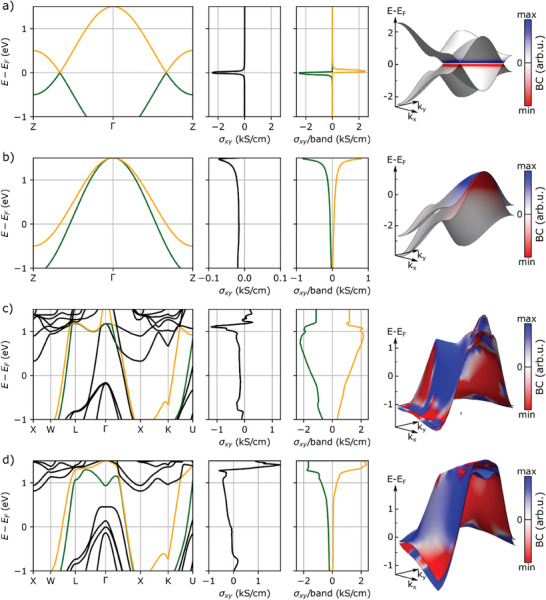
Band dependent AHC in a toy model and real compounds. Each row shows from left to right: band structure, total AHC, band‐dependent AHC, and the bands in the *k*_z_ = 0 plane with the color expressing the AHC at that point. a) Simple Weyl model considering two crossings at the Fermi energy, *E*_F_. The AHC is very pronounced at the *E*_F_ and decays rapidly as the energy shifted away. b) Weyl model with two Weyl points at *E*‐*E*_F_ = 1.4 eV. The AHC still shows a peak at the crossing energy but with a long tail to lower energies. c) NiMnSb with Weyl crossings at *E*‐*E*_F_ = 1.1 eV. d) PtMnSb with Weyl crossings at *E*‐*E*_F_ = 1.26 eV. In both compounds the colored bands form the crossings, which generate a large AHC with a long tail to lower energies similar to b).

This model describes two independent isotropic bands that are coupled by a term simulating SOC with strength A. Depending on the choice of parameters, different systems can be investigated. In **Figure** **6**a, a symmetric Weyl semimetal with two Weyl points at *E*_F_ (M_1_ = −1.5 eV, M_2_ = 1.5 eV, B_1_ = 0.5 eV, B_2_ = −0.5 eV, A = 0.01 eV) is shown. As expected for the model systems, there is a large and pronounced peak of the AHC at the energy of the crossings.^[^ ^39^ ^]^ It can be seen, that the AHC is highly localized in energy and decays rapidly when moving away from the *E*_F_. This can also be seen in the 3D band structure, where the bands carry significant AHC only around the *E*_F_. However, the two‐band model can also simulate a different situation. In Figure 6b, the two bands form crossings 1.4 eV above *E*_F_ (M_1_ = −1.5 eV, M_2_ = −2.49 eV, B_1_ = 0.5 eV, B_2_ = 0.6657 eV, A = 0.01 eV), which leads to some interesting properties. In this system model, there is still a pronounced peak at the energy of the Weyl points, but the localization in energy is much smaller compared to the first model. At lower energies, the AHC falls off very slowly and even remains constant at ≈about 20% of the peak value for a large energy range around *E*_F_. This is due to the fact that two bands run almost parallel with a large velocity down to lower energies from the crossing. As it can be seen from the 3D band structure, each band mainly carries either positive or negative AHC, leading to a partial compensation of the two contributions. However, since the bands still have an energy separation, the compensation is only partial, resulting in a very stable AHC over a wide energy range. As there are no other features in these simple tight‐binding models, we can conclude that the Weyl points at 1.4 eV above *E*_F_ induce the full AHC in the second model also at the *E*_F_.

From the findings obtained with these simple models, we can conclude that even topological features such as Weyl points located far from the *E*_F_ can influence, and even dominate, the BC‐related transport values if suitable band dispersions are present. Since the same key features were found in both NiMnSb (Figure 6c) and PtMnSb (Figure 6d), including the large peak at the Weyl points with a slowly decaying tail, the presence of two nearly parallel bands with a large velocity, and strongly opposing AHCs carried by these two bands, we can conclude that the measured AHC values in these materials are dominated by Weyl points that are located more than 1 eV above the *E*_F_. This contradicts the typical assertion that transport is only affected by states at the *E*_F_, and demonstrates the versatility and uniqueness of BC‐related transport.

## 3 Conclusion

We have investigated the electronic and magnetic transport properties and the band structure of the well‐known half‐metallic and the pseudo half‐metallic half‐Heusler ferromagnets NiMnSb and PtMnSb. Both the compounds exhibit extremely high *T*_C_ values of 660 and 560 K, respectively, with a saturation magnetization of approximately 4 µ_B_/f.u. Ab initio calculations revealed several band crossings resulting from Weyl nodes in both the compounds. More importantly, we determined that the main source of the AHC in both the compounds is the Weyl nodes that were located far from the *E*_F_, which influence the transport at the *E*_F_ due to the unique band dispersions. Our findings pave the way for future research on other half‐Heusler ferromagnets that exhibit anomalous transport owing to the presence of non‐trivial topological states. Since many materials have not been thoroughly investigated for topological nodes that are located far from *E*_F_, our study provides a foundation for future studies on other topological materials to investigate the dragging effect of the BC.

## 4 Experimental Section

### Single Crystal Growth

Single crystals of NiMnSb and PtMnSb were grown by the flux‐growth method using bismuth (Bi) as a flux. Highly purified Mn (99.999%), Pt (Ni) (99.999%), Sb (99.99%) and Bismuth rod (99.999%) were cut into small pieces and weighed in a 1:1:1:10 molar ratio, resulting in a total weight of 15 g. This stoichiometric amount was placed in an alumina crucible altogether. The alumina crucible was then sealed in a quartz tube at argon pressure of 0.2 bar. The quartz ampule was placed in box furnace and heated to 800 °C (1000 °C) at a rate of 100° C h^−1^. For homogeneity, the content was kept at this temperature for 24 hours.

The furnace temperature was slow cooled to 400 °C (600 °C), at a rate of 2 °C h^−1^, for the crystal growth. At 400 °C (600 °C), the extra flux was removed by centrifugation. From this procedure, silvery single crystals with 1−2 mm sizes were obtained, in which [111] was a preferred growth orientation. Several crystals were separated for further characterizations. The composition of NiMnSb and PtMnSb crystals was determined using scanning electron microscopy and an energy‐dispersive EDAX analyzer. The analyses results were given in Table S1 in Supporting Information.

NiMnSb was also grown by laser floating zone technique (Crystal Systems Inc., Japan). Highly purified Mn (99.999%), Ni (99.999%), and Sb (99.99%) were cut into small pieces and weighed in a 1:1:1 molar ratio, resulting in a total weight of 5 g. Three homogeneous polycrystalline ingots were prepared in arc melting furnace each of 5 g. These polycrystalline ingots (15 g) were used to prepare rod in levitation melting facility. These rods were further used as seed and feed rods to grow crystals. The rotation of the upper and lower shafts was kept at 20 rpm. The growth rate was 1 mm h^−1^, which was fixed over whole growth time period.

### Magnetization and Electrical Transport Measurements

A superconducting quantum interference device vibrating sample magnetometer (MPMS 3, Quantum Design) with a sensitivity of 10^−7^ emu was used to make the magnetic measurements. The field as well as temperature‐dependent magnetization for both compounds were measured over a magnetic field range of −7 to +7 T at temperatures between 2 and 800 K. The ETO with rotator option was used to measure resistivity in a physical property measurement system (PPMS‐9T; Quantum Design). Using a wire saw, samples with bar shapes of desired crystallographic orientations were cut from single crystals. Laue X‐ray diffraction were performed to confirm the orientation of these crystals. Silver paint and 25 µm platinum wires were used to make standard 4 probe contacts on the oriented crystals respectively, using a current of 8.0 mA for all temperatures in range of 2– 300 K. The Hall resistivity was derived by anti‐symmetrizing in order to reduce longitudinal resistivity contribution owing to voltage probe misalignment.

### Theoretical Methods

For investigating the compound, the ab initio method of Density‐Functional Theory (DFT) was utilized via the VASP‐package.^[^ ^42^ ^]^ The exchange‐correlation potential corresponds to that of the Generalized Gradient Approximation (GGA).^[^ ^41^ ^]^ In this procedure, a grid of 10 × 10 ×  10, with a precision of 10^−6^eV has been used, leading to a magnetic moment of: µ  =  4.04µ_*B*_ and µ  =  4.06µ_*B*_ for NiMnSb and PtMnSb, respectively, in coherence with previous literature^[^ ^44^ ^]^ and the experimental results. The magnetization has been computed in the (1,1,1) direction to match the experimental set up. In the next step, a wannierization process was implemented via the software WANNIER90,^[^ ^45^ ^]^ employing *s, p* and *d* projections for each one of the atoms in both compounds. From it, a tight‐binding Hamiltonian can be derived, leading to an expression of the tensor of the Berry curvature in reciprocal space in terms of velocity matrix elements, as^[^ ^46^ ^]^:

(2) $$\Omega_{ij}^{m}\;\left(\vec{k}\right)=\;i\;\sum_{m\neq n}\frac{v_{i}^{mn}v_{j}^{nm}-\left(i↔j\right)}{{\left(E_{m}-E_{n}\right)}^{2}}\;$$

where: $v_{\mu }^{mn}\;\left(\vec{k}\right)=\frac{1}{\hbar }\;\langle m|\frac{\partial \hat{H}}{\partial k_{\mu }}\left|n\right\rangle$. Making use of Kubo formalism at T = 0 K, one has the anomalous Hall conductivity (AHC):^[^ ^46^ ^]^

(3) $$\begin{array}{ccc} \sigma_{ij} & = & e^{2}\;\hbar \;\sum_{m}^{occ.}\sum_{m\neq n}\int \frac{d^{3}k}{{\left(2\pi \right)}^{3}}\frac{v_{i}^{mn}\left(\vec{k}\right)v_{j}^{nm}\left(\vec{k}\right)-\left(i↔j\right)}{{\left(E_{m}-E_{n}\right)}^{2}}\\ & = & e^{2}\;\hbar \sum_{m}^{occ}\int \frac{d^{3}k}{{\left(2\pi \right)}^{3}}\Omega_{ij}^{m}\left(\vec{k}\right) \end{array}$$

This implementation for the AHC was done with an internal program of the group.

## Conflict of Interest

The authors declare no conflict of interest.

## Supporting information

Supporting Information

## Data Availability

The data that support the findings of this study are available from the corresponding author upon reasonable request.
