# Supplementary material for: Extended Berry Curvature Tail in Ferromagnetic Weyl Semimetals NiMnSb and PtMnSb
Source: Adv Sci (Weinh). 2024 Jun 18;11(31):2404495. doi: 10.1002/advs.202404495 (PMC11336927; doi:10.1002/advs.202404495)
Supplement: Supplementary file 1 — Supporting Information [file ADVS-11-2404495-s001.pdf]

ADVANCED SCIENCE
Open Access

Supporting Information

for *Adv. Sci.*, DOI 10.1002/advs.202404495

Extended Berry Curvature Tail in Ferromagnetic Weyl Semimetals NiMnSb and PtMnSb

Sukriti Singh, Ana García-Page, Jonathan Noky, Subhajit Roychowdhury, Maia G. Vergniory, Horst Borrmann, Hans-Henning Klauss, Claudia Felser and Chandra Shekhar**

Supporting Information

Extended Berry curvature tail in ferromagnetic Weyl semimetals NiMnSb and PtMnSb

*Sukriti Singh, Ana García-Page, Jonathan Noky, Subhajit Roychowdhury, Maia G. Vergniory, Horst Borrmann, Hans-Henning Klauss, Claudia Felser, and Chandra Shekhar**

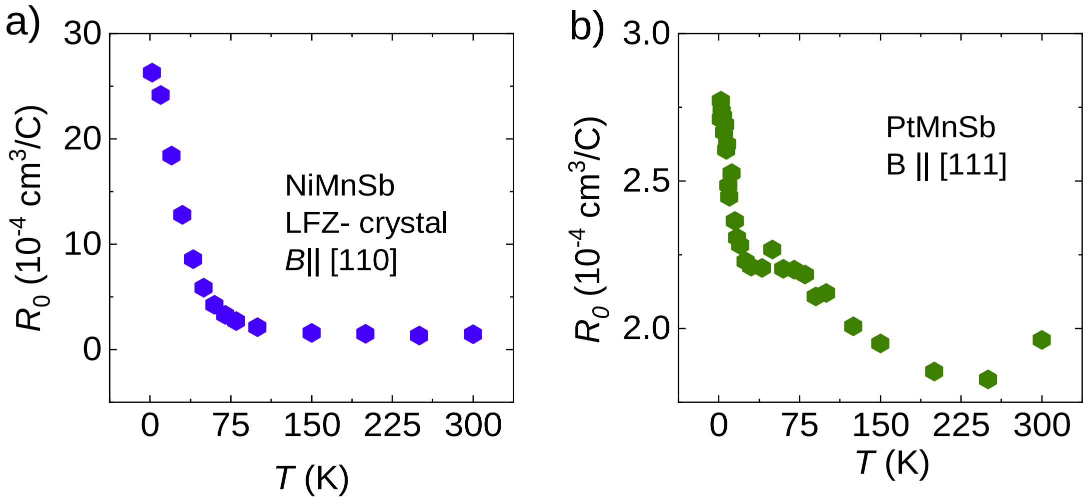

Figure S1: Hall coefficient R_0 Temperature dependence of Hall coefficient R_0 of **a)** NiMnSb and **b)** PtMnSb respectively.

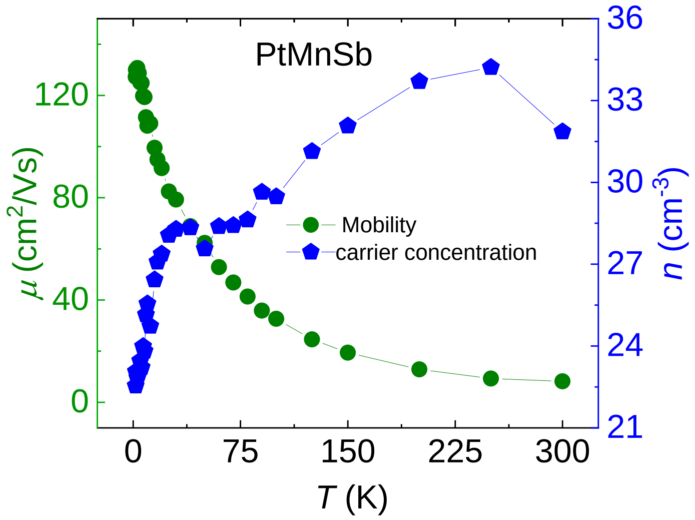

Figure S2: Temperature dependent mobility and carrier concentration of PtMnSb.

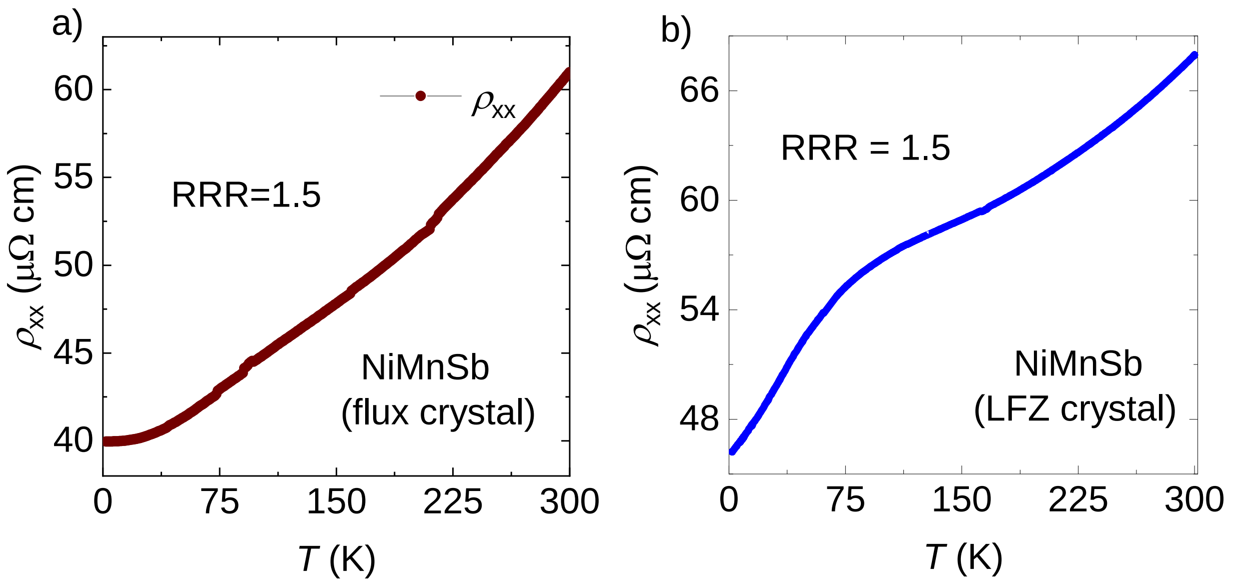

Figure S3: Temperature dependent longitudinal resistivity of NiMnSb a) flux crystal b) LFZ crystal

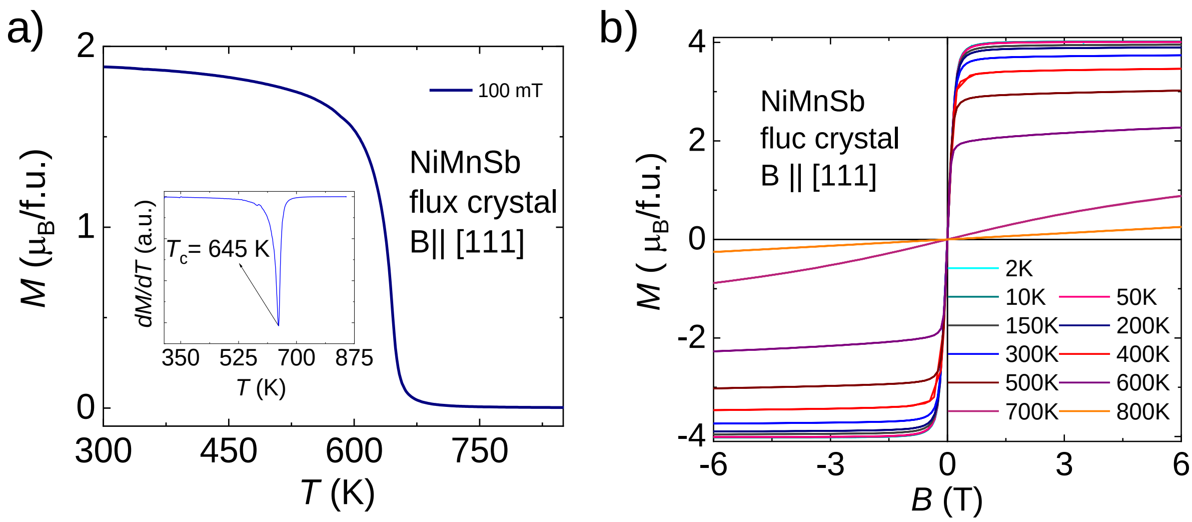

Figure S4: Magnetization of NiMnSb flux crystal a) Temperature dependent magnetization measured at applied magnetic field of 100 mT along [111] direction. Inset shows first order derivative of magnetization where peak shows Curie temperature T_C of 645 K. b) Field dependent magnetization curve.

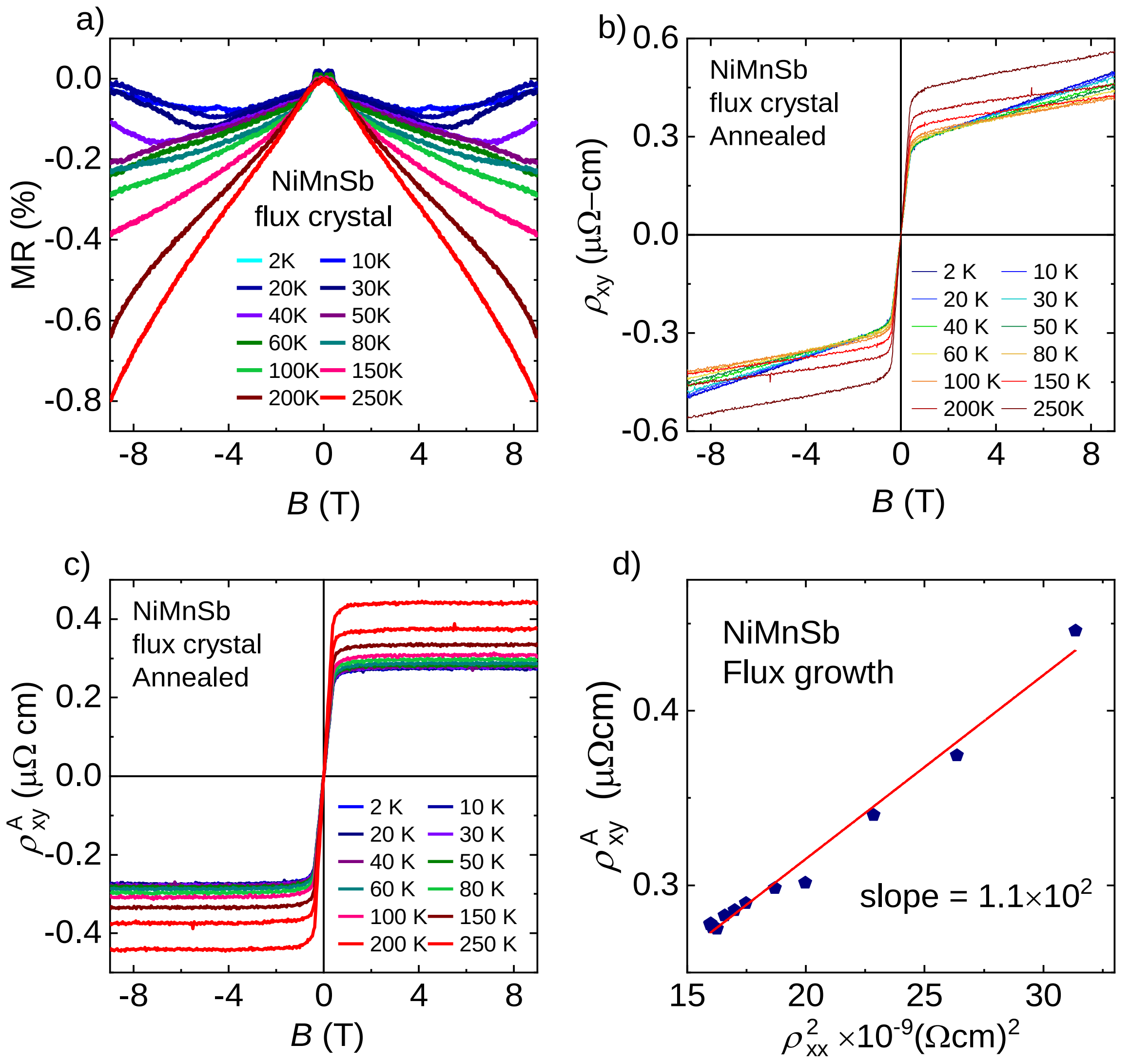

Figure S5: Transverse and Hall resistivity a) MR of flux crystal measured at various temperature in range of 2- 300 K shows negative MR b) Field dependent Hall resistivity c) Reduced anomalous Hall resistivity with field d) Linear fit on plot of anomalous Hall resistivity and longitudinal resistivity, where slope gives contribution from Berry curvature.

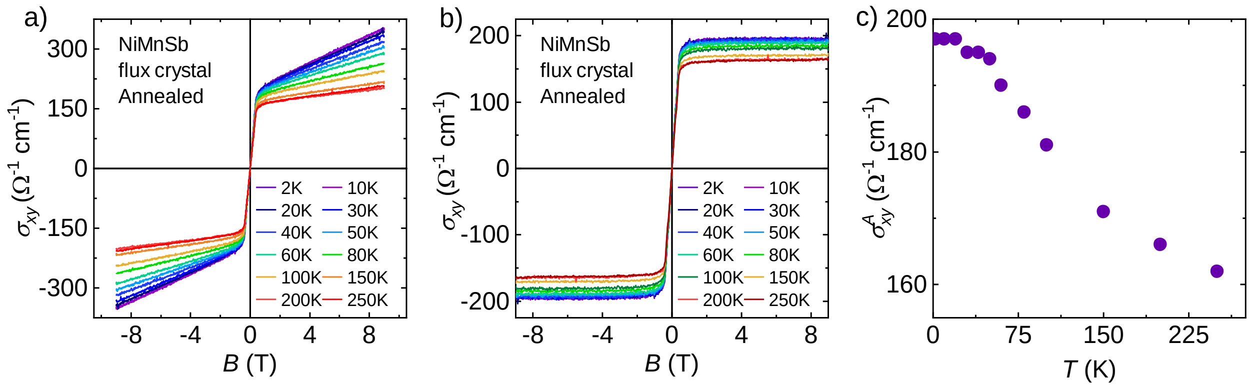

Figure S6: Anomalous Hall conductivity of NiMnSb flux crystal a) Field dependent Hall conductivity with applied field along [111] direction. b) Anomalous Hall conductivity reduced by subtracting ordinary Hall effect c) Temperature dependence AHC

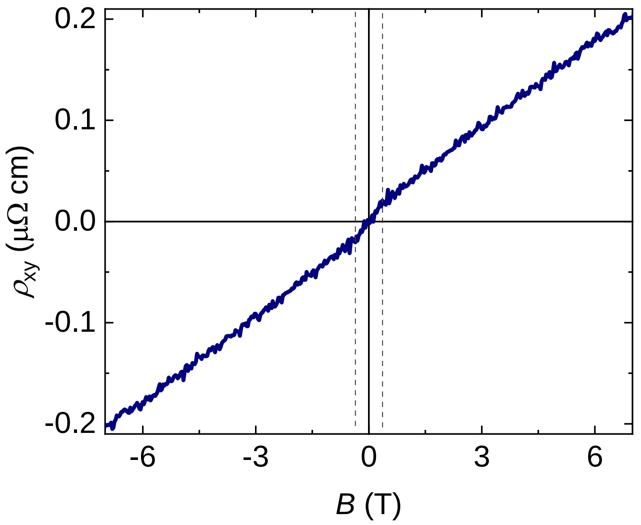

Figure S7: Field dependent Hall resistivity of PtMnSb at 2 K

Table S1: EDAX analysis of NiMnSb LFZ and flux crystal

NiMnSb Flux crystal

EDAX ZAF Quantification (Standardless)
Element Normalized
SEC Table : User c:\edax32\eds\genuser.sec

Elem	Wt %	At %	K-Ratio	Z	A	F
SbL	50.87	32.56	0.4807	0.9263	1.0086	1.0114
MnK	24.49	34.73	0.2344	1.0526	0.8915	1.0203
NiK	24.64	32.70	0.2498	1.0982	0.9233	1.0000
Total	100.00	100.00				

Element	Net Inte.	Backgrd	Inte. Error	P/B
SbL	1836.10	1.80	0.52	1020.06
MnK	1187.10	0.75	0.65	1582.80
NiK	771.85	0.80	0.81	964.81

NiMnSb LFZ crystal

Elem	Wt %	At %	K-Ratio	Z	A	F
SbL	48.87	30.83	0.4610	0.9238	1.0090	1.0120
MnK	25.60	35.79	0.2454	1.0494	0.8944	1.0213
NiK	25.52	33.39	0.2580	1.0947	0.9235	1.0000
Total	100.00	100.00				

Element	Net Inte.	Backgrd	Inte. Error	P/B
SbL	1777.25	3.70	0.53	480.34
MnK	1255.80	5.25	0.63	239.20
NiK	805.75	4.00	0.79	201.44

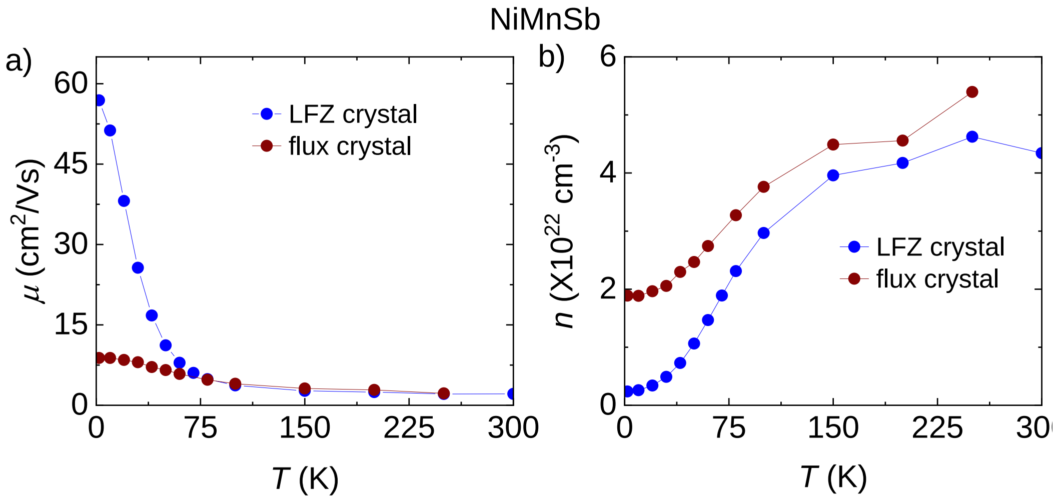

Figure S8: a) Mobility and b) carrier concentration of NiMnSb LFZ crystal and flux crystal

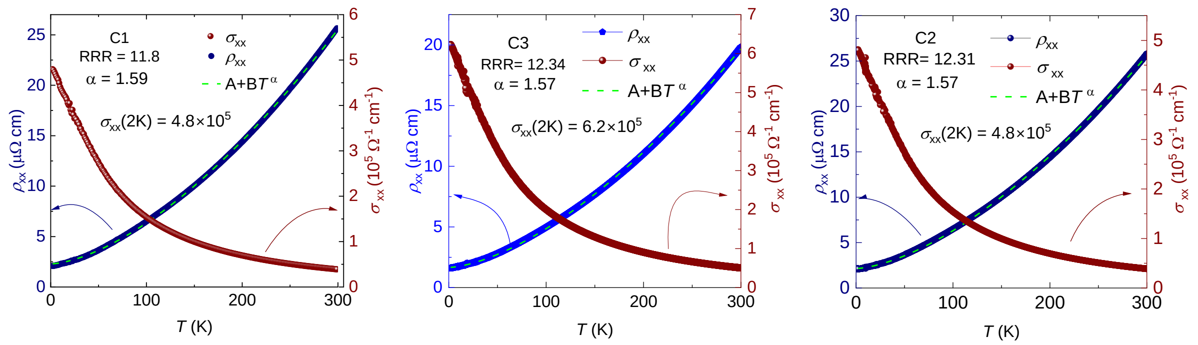

Figure S9: Longitudinal resistivity and conductivity measured for three different crystals of PtMnSb and their temperature dependent resistivity fit.

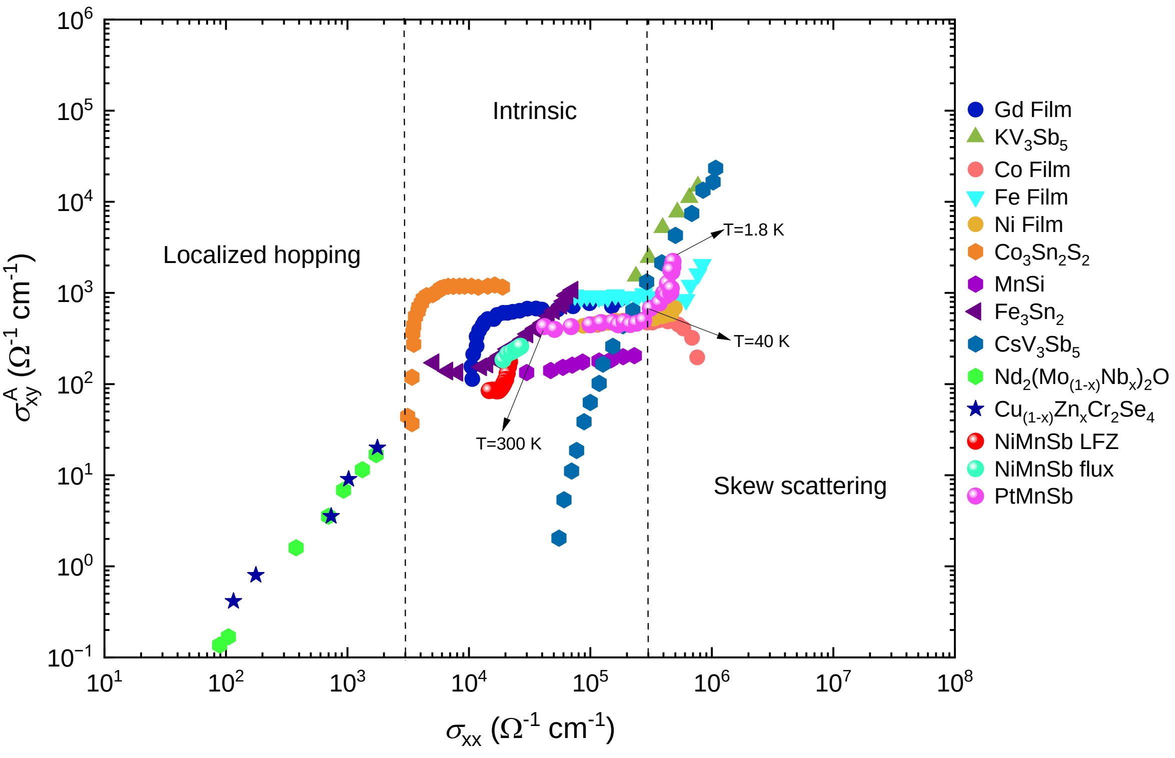

Figure S10: σ_{xy}^{A} versus σ_{xx} for a variety of materials depicting the different regimes of localized hopping, intrinsic (Berry Curvature) and skew scattering contribution.

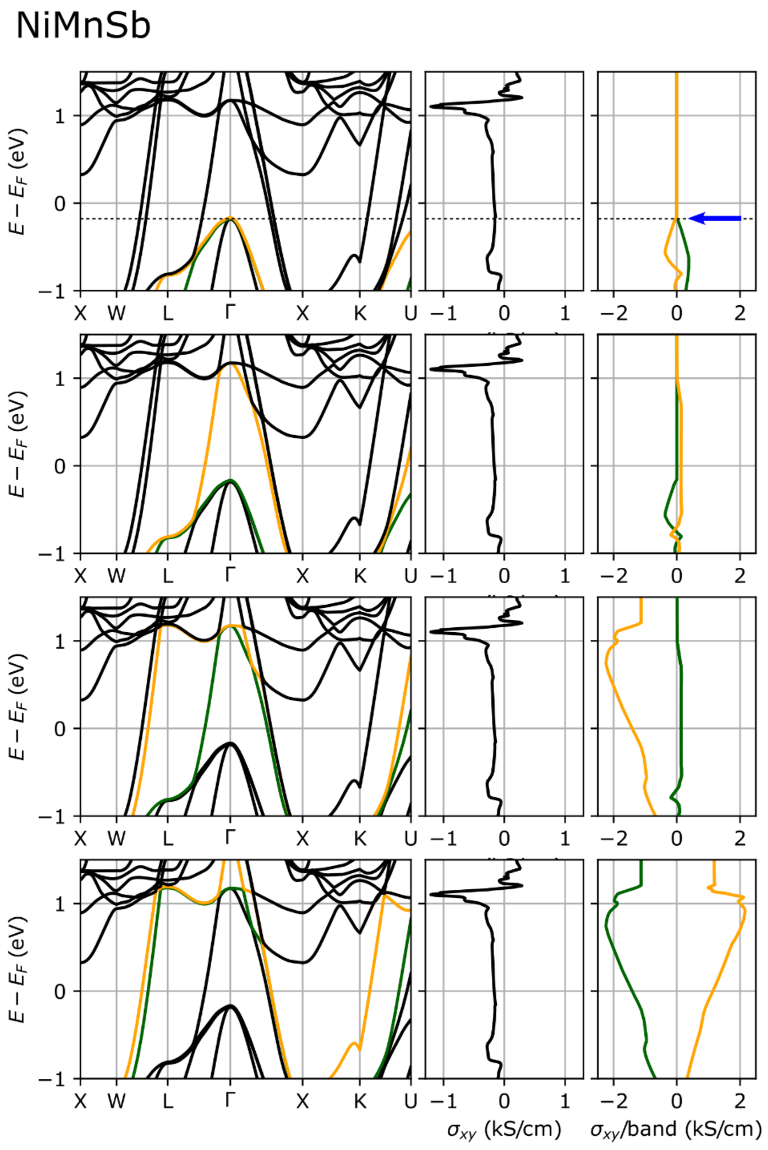

Figure S11: Band-dependent AHC of all bands crossing the Fermi level in NiMnSb. For each row, from left to right: Band structure with the two analyzed bands highlighted; total AHC; band-dependent contributions of the highlighted bands. The Weyl crossings close to the Fermi level are marked in the respective row with a dashed line and a blue arrow. If can be seen, that these crossings have a negligible contribution to the total AHC. Even more general, the contributions of the two bands forming the crossings far above the Fermi level have much larger contributions than all other bands in the whole shown energy range. Therefore, we can conclude, that these far away crossings are the main source of intrinsic AHC even at the Fermi level in the NiMnSb.

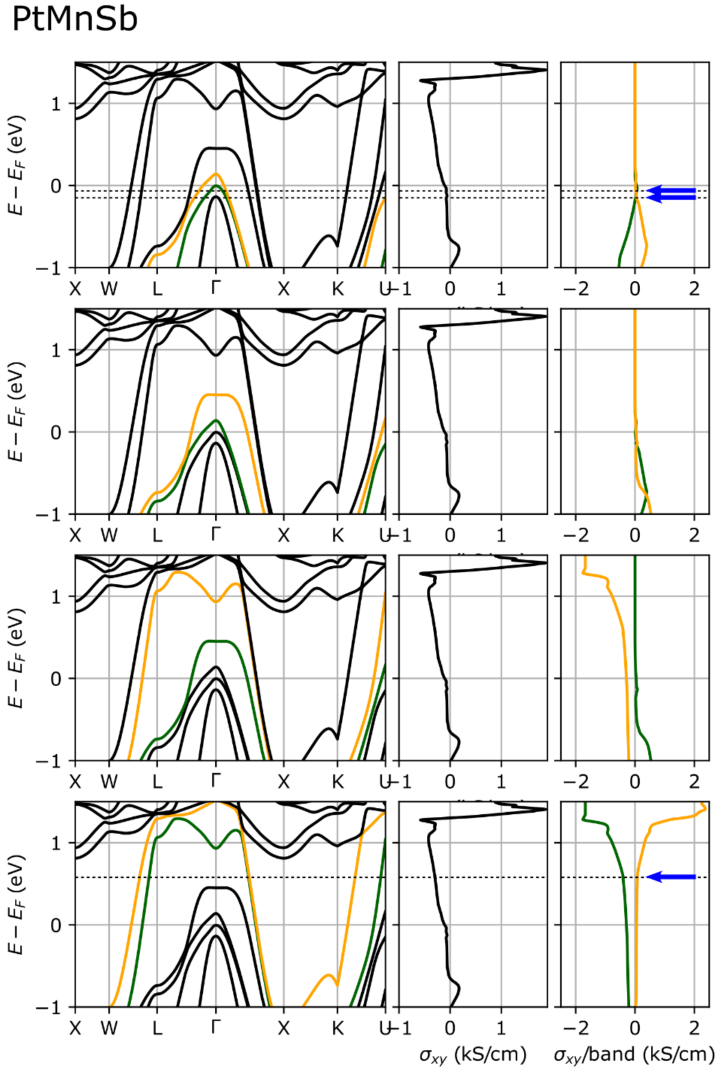

Figure S12: Band-dependent AHC of all bands crossing the Fermi level in PtMnSb. For each row, from left to right: Band structure with the two analyzed bands highlighted; total AHC; band-dependent contributions of the highlighted bands. The Weyl crossings close to the Fermi level are marked in the respective row with a dashed line and a blue arrow. If can be seen, that these crossings have a negligible contribution to the total AHC. Even more general, the contributions of the two bands forming the crossings far above the Fermi level have much larger contributions than all other bands in the whole shown energy range. Therefore, we can conclude, that these far away crossings are the main source of intrinsic AHC even at the Fermi level in the PtMnSb.

Table S2. Position of some of the Weyl nodes for NiMnSb.

$k_x\ (1/a)$	$k_y(1/b)$	$k_z\ (1/c)$	$E-E_F\ \ (eV)$
0.0025	0.0025	0.0025	-0.174
-0.0025	-0.0025	-0.0025	-0.174
0.0171	0.0171	0.0171	1.112
-0.0171	-0.0171	-0.0171	1.112
0.0960	0.0578	-0.0670	1.112
-0.0626	-0.1002	-0.0684	1.112
0.1004	0.0628	0.0685	1.112
-0.1003	-0.0685	-0.0627	1.112
-0.0960	0.0669	-0.0579	1.112
0.0575	-0.0669	0.0959	1.112
0.0628	0.0685	0.1004	1.112
-0.0683	-0.0627	-0.1002	1.112
-0.0669	0.0959	0.0577	1.112
0.0684	0.1003	0.0627	1.112
0.0667	-0.0576	-0.0958	1.112
-0.0577	-0.0959	0.0668	1.112

Table S3. Position of some of the Weyl nodes for PtMnSb

$k_x\ (1/a)$	$k_y(1/b)$	$k_z\ (1/c)$	$E - E_F\ \ (eV)$
-0.0347	-0.0347	-0.0347	-0.0695
0.0347	0.0347	0.0347	-0.0695
0.0447	0.0485	-0.0117	-0.136
0.0484	-0.0118	0.0447	-0.136
-0.0448	0.0117	-0.0485	-0.136
-0.0117	0.0447	0.0485	-0.136
0.0117	-0.0484	-0.0447	-0.136
-0.0484	-0.0448	0.0118	-0.136
0.0247	0.0250	-0.0544	0.624
-0.0251	-0.0249	0.0544	0.624
0.0251	-0.0543	0.0249	0.624
-0.0251	0.0544	-0.0252	0.624
-0.0544	0.0251	0.0253	0.624
0.0544	-0.0253	-0.0252	0.624
0.0739	0.0809	-0.0831	1.26
-0.0809	-0.0738	0.0831	1.26
0.0608	-0.0307	0.0317	1.26
0.0809	-0.0831	0.0739	1.26
-0.0739	0.0831	-0.0809	1.26
-0.0608	-0.0316	0.0306	1.26
0.0314	0.0608	-0.0305	1.41
-0.0304	0.0314	0.0608	1.41
0.0702	0.0702	0.0702	1.41
0.0534	0.0534	0.0534	1.41
-0.0702	-0.0702	-0.0702	1.41
-0.0831	0.0739	0.0809	1.41
0.0831	-0.0809	-0.0739	1.41
0.0307	-0.0608	-0.0317	1.41
-0.0315	0.0305	-0.0608	1.41
